# Combined Pharmacological Inhibition of Cyclophilins, FK506-Binding Proteins, Hsp90, and Hsp70 Protects Cells From *Clostridium botulinum* C2 Toxin

**DOI:** 10.3389/fphar.2018.01287

**Published:** 2018-11-13

**Authors:** Katharina Ernst, Carolin Kling, Marc Landenberger, Holger Barth

**Affiliations:** Institute of Pharmacology and Toxicology, Ulm University Medical Center, Ulm, Germany

**Keywords:** bacterial protein toxin, PPIases, cyclophilins, FK506 binding proteins, chaperones, membrane translocation, protein interaction, specific pharmacological inhibition

## Abstract

The *Clostridium botulinum* C2 toxin is an exotoxin causing severe enterotoxic symptoms. The C2 toxin consists of the binding/translocation component C2II, and the enzymatic active component C2I. After proteolytic activation, C2IIa forms heptamers that bind C2I. The C2IIa/C2I complex is taken up into mammalian target cells via receptor-mediated endocytosis. Acidification of endosomes leads to conformational changes in both components. C2IIa heptamers form a pore into the endosomal membrane, and C2I becomes unfolded and translocates through the narrow C2IIa pores into the cytosol of the cell. Here, C2I covalently transfers an ADP-ribose moiety from its co-substrate NAD^+^ onto G-actin, which leads to depolymerization of F-actin resulting in rounding up of adherent cells. Translocation of C2I into the cytosol depends on the activity of the chaperones Hsp90 and Hsp70 and peptidyl-prolyl *cis/trans* isomerases of the cyclophilin (Cyp) and FK506-binding protein (FKBP) families. Here, we demonstrated that C2I is detected in close proximity with Hsp90, Cyp40, and FKBP51 in cells, indicating their interaction. This interaction was dependent on the concentration of C2 toxin and detected in mammalian Vero and human HeLa cells. Moreover, the present study reveals that combination of radicicol, VER-155008, cyclosporine A, and FK506, which are specific pharmacological inhibitors of Hsp90, Hsp70, Cyps, and FKBPs, respectively, resulted in a stronger inhibition of intoxication of cells with C2 toxin compared to application of the single inhibitors. Thus, the combination of inhibitors showed enhanced protection of cells against the cytotoxic effects of C2 toxin. Cell viability was not significantly impaired by application of the inhibitor combination. Moreover, we confirmed that the combination of radicicol, VER-155008, CsA, and FK506 in particular inhibit the membrane translocation step of C2I into the cytosol whereas receptor binding and enzyme activity of the toxin were not affected. Our findings further characterize the mode of action of Hsp90, Hsp70, Cyps, and FKBPs during membrane translocation of bacterial toxins and furthermore supply starting points for developing of novel therapeutic strategies against diseases caused by bacterial toxins that depend on Hsp90, Hsp70, Cyps, and FKBPs.

## Introduction

The *Clostridium botulinum* C2 toxin is a bacterial exotoxin and represents the prototype of the family of clostridial binary toxins which comprises amongst others the *C. perfringens* iota toxin and the *C. difficile* CDT toxin (Barth and Aktories, 2011; Stiles, 2017). These toxins are secreted by the respective bacteria and consist of two non-linked proteins, the binding/translocation B-component, and the enzymatically active A-component. The B-component binds to a specific receptor on target cells and mediates the uptake of the A-component via receptor-mediated endocytosis. The B-component forms a pore into the endosomal membrane through which the A-component translocates into the cytosol. Here, the A-component covalently transfers an ADP-ribose moiety onto monomeric actin (G-actin), which leads to a depolymerization of the actin cytoskeleton and therefore to rounding of target cells (Reuner et al., 1987; Aktories and Wegner, 1992; Aktories et al., 2017b). All three toxins cause severe enterotoxic symptoms in humans or animals, which are the consequence of their enzymatic mode of action in cells. The C2 toxin causes necrosis and hemorrhagic lesions in the intestinal mucosa of mice (Simpson, 1982; Ohishi, 1983a,b) and fluid accumulation in the intestinal loop of pheasants and chicken (Kurazono et al., 1987). For the iota toxin, lambs and calves have been identified as common casualties for its enterotoxicity (Songer, 1996; Billington et al., 1998). *C. difficile* infections (CDI) are still on the rise in hospitals of Western countries and pose a severe threat due to life-threatening symptoms such as antibiotic-associated diarrhea or pseudomembranous colitis. CDT has been identified as a novel virulence factor produced by hypervirulent *C. difficile* strains and most likely contributes to an improved colonization of *C. difficile* in the human gut (Aktories et al., 2018; Papatheodorou et al., 2018).

The prototype of clostridial toxins, C2 toxin is composed of the A-component C2I and the B-component C2II (Ohishi, 1983a,b). After proteolytic activation of C2II, the resulting C2IIa forms ring-shaped heptamers that bind to carbohydrate structures, which have been found on the surface of all cell types, investigated so far (Barth et al., 2000; Eckhardt et al., 2000). C2I attaches to specific motifs of the C2IIa heptamer and the C2IIa/C2I complex is taken up via receptor-mediated endocytosis (Barth et al., 1998a; Blöcker et al., 2000; Kaiser et al., 2006). Acidification of the endosomal lumen results in formation of a C2IIa pore with a narrow inner diameter of 1–2 nm into the endosomal membrane (Barth et al., 2000; Schleberger et al., 2006). At least partial unfolding of C2I is required to translocate through the narrow C2IIa pore into the target cell cytosol where it ADP-ribosylates G-actin (Aktories et al., 1986; Haug et al., 2003b). We demonstrated earlier that translocation of C2I into the cytosol is facilitated not only by the C2IIa pore but requires activity of host cell chaperones and peptidyl-prolyl *cis/trans* isomerases (PPIases) [for review see (Schiene-Fischer, 2015; Barth and Ernst, 2016; Ernst et al., 2017b; Schopf et al., 2017)]. We identified the heat shock protein Hsp90 and Hsp70 as well as isoforms of the cyclophilin (Cyp) and FK506 binding protein (FKBPs) family, namely CypA, Cyp40, and FKBP51, as specific interaction partners for C2I. Hsp90 and Hsp70 activities are ATP-dependent and play important roles during several cellular processes such as folding, refolding, avoiding aggregation of unfolded proteins as well as protein transport, e.g., through the endoplasmic reticulum or into mitochondria (Freeman and Morimoto, 1996; Chacinska et al., 2009; Clerico et al., 2015; Schopf et al., 2017). Cyps and FKBPs catalyze the *cis/trans* isomerization of prolyl-bonds, which often represents a rate-limiting step in protein folding (Brandts et al., 1975; Schiene-Fischer, 2015). Specific pharmacological inhibition of Hsp90, Hsp70, Cyps, and FKBPs activities prevented translocation of C2I into the cytosol and protected cells from intoxication. Direct interaction between chaperones/PPIases and C2I was demonstrated and characterized *in vitro* by various methods including dot blot analysis, co-precipitation, and isothermal titrations calorimetry (Haug et al., 2003a, 2004; Kaiser et al., 2009, 2011, 2012; Ernst et al., 2015, 2016, 2017a). Here, we demonstrate the interaction of Hsp90, Cyp40, and FKBP51 with C2I in cells by employing a fluorescence-based proximity ligation assay (PLA). Moreover, we discovered that the simultaneous application of the various specific pharmacological inhibitors of Hsp90, Hsp70, Cyps, and FKBPs resulted in a significantly stronger inhibitory effect on C2 intoxication compared to the single application of each inhibitor. Moreover, the combined pharmacological inhibition of the chaperones/PPIases in cells resulted in a stronger inhibition of the membrane translocation of C2I into the cytosol compared to inhibition of each host cell factor alone. This might lead to novel pharmacological strategies to treat/prevent the severe enteric diseases in humans and animals that are associated with binary clostridial toxins.

## Materials and Methods

### Protein Expression and Purification

The recombinant proteins C2I, C2I E389/387Q, C2IIa were purified and activated as described before (Barth et al., 1998b). In brief, recombinant proteins were overexpressed as GST-fusion proteins in *E. coli* BL21 cells, batch-purified and the GST-tag was cleaved with thrombin. C2IIa was activated with trypsin.

### Cell Culture and Intoxication Experiments

Vero (African green monkey kidney) and HeLa cells (from DSMZ, Braunschweig, Germany) were cultured in MEM plus 10% heat-inactivated fetal calf serum (FCS) (GIBCO^®^ life technologies, Karlsruhe, Germany), 0.1 mM non-essential amino acids, 1 mM sodium pyruvate, 2 mM L-glutamine, and 10% penicillin–streptomycin at 37°C and 5% CO_2_. Cells were detached using trypsin and 25 cycles of reseeding were performed. For intoxication experiments, cells were seeded in 24-well culture dishes and incubated with C2I and C2IIa at 37°C and 5% CO_2._ To analyze the effect upon inhibition of host cell factors, cells were pre-incubated with the specific inhibitors cyclosporine A (CsA, inhibitor of Cyp activity, Sigma-Aldrich, Steinheim, Germany), FK506 (inhibitor of FKBP activity, Sigma-Aldrich, Steinheim, Germany), radicicol (Rad, inhibitor of the ATP-binding site of Hsp90, Sigma-Aldrich, Steinheim, Germany), and VER-155008 (VER, inhibitor of the ATP-binding site of Hsp70 and Hsc70, Sigma-Aldrich, Steinheim, Germany) 30 min prior to toxin application. Pictures of cells were taken using a Zeiss Axiovert 40CFL microscope with a Jenoptik ProGres C10 CCD camera. Toxin-specific morphological changes (i.e., cell rounding) were analyzed by determining the percentages of rounded cells by manually counting the cells per picture using ImageJ (National Institutes of Health, Bethesda, United States). Materials for cell culture were purchased from TPP Techno Plastic Products. Cell viability was measured by using MTS [3-(4,5-dimethylthiazol-2-yl)-5-(3-carboxymethoxyphenyl)-2-(4-sulfo phenyl)-2H-tetrazolium] cyto-toxicity assay Cell Titer 96 Aqueous from Promega (Mannheim, Germany) according to the manufacturer’s instructions.

### Immunofluorescence

Cells were seeded in ibidi μ-slide eight-well plates. After respective treatments, cells were washed and fixed with 4% paraformaldehyde (PFA) and 100% ice-cold methanol. After permeabilization with 0.4% Triton X-100, quenching with 100 nM glycine, and blocking with 10% FCS, anti-C2IN serum, and subsequently fluorescence labeled secondary antibody were added. Images were obtained using iMic digital microscope (FEI Munich) and Live Acquisition 2.6 software (FEI Munich) and were processed with ImageJ software (National Institutes of Health, Bethesda).

### Analysis of Protein Interaction Using PLA Technology

Cells were seeded in ibidi μ-slide eight-well plates and incubated with C2 toxin for 1 h at 37°C, washed with PBS and then fixed with 4% PFA in PBS for 15 min followed by freezing methanol for 1 min. Cells were permeabilized with Triton X-100 (0.4% in PBS) for 5 min, treated with 100 mM glycine in PBS, and blocked with blocking solution (Duolink using PLA technology, Sigma-Aldrich, St. Louis, United States) for 1 h at 37°C. Cells were incubated with rabbit anti C2IN (N-terminal part of C2I) serum (1:1500) and mouse anti Hsp90 antibody (1:8000) (Santa Cruz Biotechnology, Dallas, United States), mouse anti FKBP51 antibody (1:100) (Abnova, Taipei City, Taiwan), or mouse anti Cyp40 antibody (1:100) (Acris Antibodies GmbH, Herford, Germany), all diluted in antibody diluent (Duolink using PLA technology, Sigma-Aldrich, St. Louis, United States) for 1 h at 37°C. Subsequently, the PLA was performed according to the manufacturer’s protocol (Duolink using PLA technology, Sigma-Aldrich), as previously described (Ernst et al., 2017a). In brief, PLA secondary antibodies contain small oligonucleotide sequences, which can form a ring structure if they get in close proximity. By addition of ligase and polymerase, a rolling circle amplification can occur. Fluorescently labeled oligonucleotides that are complementary to the amplification product allow the detection of the two target proteins, which are situated in close proximity i.e., interact with each other. In addition, fluorescence-labeled secondary antibodies were added to detect Hsp90, FKBP51, and Cyp40 in these samples.

### Analysis of Protein Levels by Western Blotting

After incubation of cells in the presence or absence of C2^∗^ for indicated time periods, cells were washed with PBS and then harvested in 2.5× Laemmli sample buffer. Samples were incubated for 10 min at 95°C, subjected to SDS–PAGE, and blotted onto a nitrocellulose membrane. Ponceau S staining was performed to confirm protein transfer and comparable protein loading. After blocking the membrane with 5% nonfat dry milk in PBS containing 0.1% Tween-20 (PBS-T), membrane was cut to allow simultaneous detection of Hsp90 (Santa Cruz, Heidelberg, Germany), FKBP51 (Bethyl Montgomery, United States) and Cyp40 (Thermo Scientific, Waltham, United States) via specific primary antibodies and secondary antibodies (Santa Cruz, Heidelberg, Germany) and enhanced chemiluminescence (ECL) system. The membrane was stripped and Hsp70 (Enzo Life Sciences, Lörrach, Germany) was detected as described above.

### Flow Cytometry

Vero cells were detached from culture dishes with 10 mM EDTA in PBS, washed twice with PBS, and then were resuspended in MEM + FCS with or without the combination of Rad, CsA, FK506, and VER. Typically, 2 × 10^5^ cells per sample were used. Cells were incubated for 30 min at 37°C and subsequently for 5 min on ice. C2IIa plus either C2I-DyLight488 or C2I^∗^-DyLight488 were added for 10 min to allow binding. Cells were washed with PBS and were then subjected to flow cytometry using a BD FACSCelesta flow cytometer and the BD FACSDiva^TM^ software. DyLight488 excitation was performed with a blue laser (488 nm) and emitted fluorescence detected with a 530 nm (530/30) bandpass filter. For data analysis and generation of fluorescence histograms from gated cell populations Flowing Software v2.5.1 (Perttu Terho, Turku Centre for Biotechnology, Finland) was used. Line smoothing (level 3) was applied to fluorescence histograms. Proteins were labeled with DyLight488 according to the manufacturer’s recommendations (Thermo Scientific, Waltham, United States). Excess dye was removed with Micro Bio-Spin 6 columns (Bio-Rad Laboratories, Munich, Germany).

### Toxin Translocation Assay

This assay was performed as described earlier (Barth et al., 2000). In short, cells were pre-incubated with the respective chaperone/PPIase inhibitors in bafilomycin A1 (BafA1, inhibitor of v-ATPase, Calbiochem^®^, Bad Soden, Germany)-containing medium. BafA1 prevents the normal toxin uptake via endosomal route. Then, cells were incubated on ice and toxin was added for 20 min to allow binding but inhibit internalization. Cells were exposed to warm acidic medium for 5 min at 37°C, which leads to pore formation by C2IIa directly into the cytoplasmic membrane and translocation of C2I through the C2IIa pore into the host cell cytosol. As control, cells were treated with neutral medium. Medium was removed and replaced by fresh BafA1-containing medium. Cell morphology was observed as a specific endpoint of intoxication.

### Analysis of *in vitro* Enzyme Activity

Vero cell lysate (20 μg of protein) was pre-incubated for 30 min at 37°C with Rad, CsA, FK506 and VER or left untreated for control. C2I and 10 μM biotin-NAD^+^ were added for 20 min at 37°C. ADP-ribosylated, i.e., biotin-labeled – G-actin was detected by Western blotting using streptavidin–peroxidase.

## Results

### C2I Is Found in Close Proximity With Hsp90, Cyp40, and FKBP51 in Cells

**FIGURE 1:**
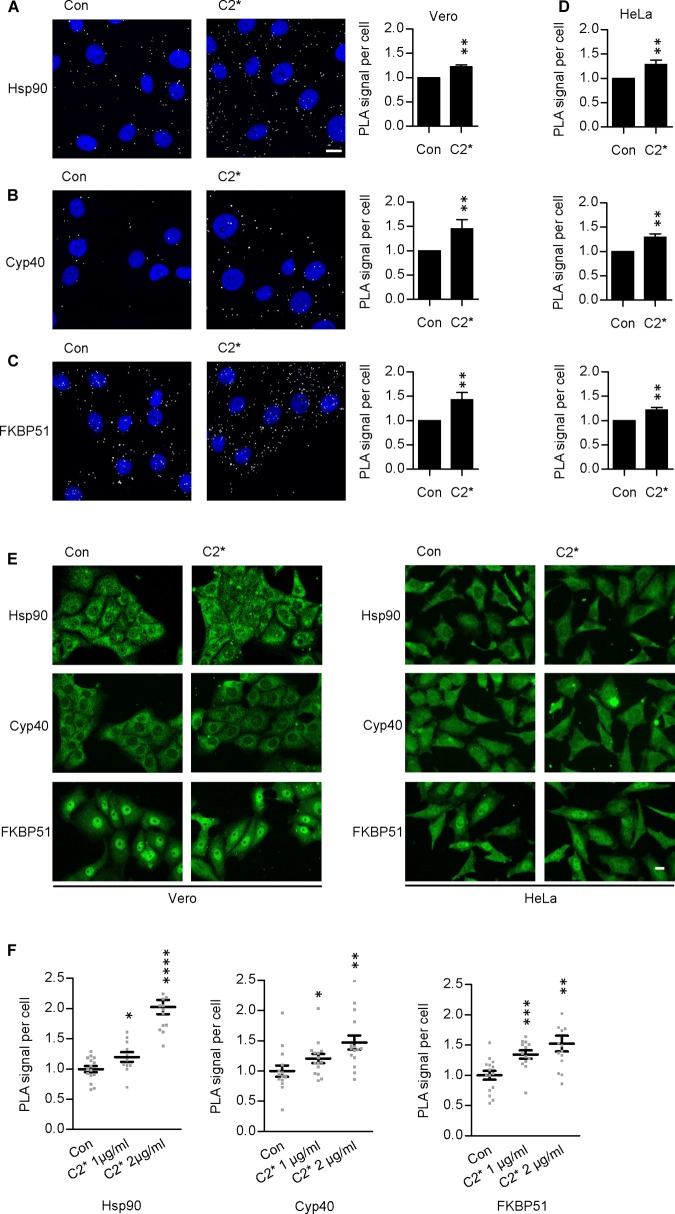
Hsp90, Cyp40, and FKBP51 are in close proximity with C2I in the cytosol of Vero cells. Vero cells were incubated with C2^∗^ toxin [C2I E387/389Q plus C2IIa, 1 μg/ml **(B)**, or 2 μg/ml **(A,C)** each] or left untreated for control for 1 h at 37°C. Subsequently, cells were fixated with 4% PFA for 15 min and 100% methanol for 1 min, blocked with Sigma blocking solution, and treated with rabbit α-C2IN (1:1500) and mouse α-Hsp90 (1:8000) **(A)**, mouse α-Cyp40 (1:100) **(B)**, or mouse α-FKBP51 (1:100) **(C)**. Then, the fluorescence-based PLA was performed according to the manufacturer’s manual. The nucleus was stained with Hoechst. Images show overlay of PLA signal (gray) and nucleus (blue). Bar = 20 μm. The amount of PLA signals per cell was quantified by ImageJ. Values are normalized to control samples and are given as mean ± SEM (*n* = 5 independent experiments, at least 200 cells per sample per experiment were analyzed). Significance was tested by using the Mann–Whitney test with GraphPad software (^∗∗^*p* < 0.01). **(D)** HeLa cells were treated with C2^∗^ toxin (C2I E387/389Q plus C2IIa, 1 μg/ml each) or left untreated for control for 1 h at 37°C. Then, cells were treated as described in **(A–C)**. **(E)** In addition to detecting PLA signals in samples **(A–D)**, Hsp90, Cyp40, and FKBP51 were visualized with fluorescence-labeled secondary antibodies in the same samples. Bar = 20 μm. **(F)** The interaction of C2I with host cell factors is concentration-dependent. Vero cells were incubated with C2^∗^ toxin (C2I E387/389Q plus C2IIa, 1 or 2 μg/ml each) or left untreated for control for 6 h at 37°C (Hsp90) or 1 h at 37°C (Cyp40, FKBP51). Subsequently, cells were treated as described above. Values are normalized to control samples and are given as mean ± SEM (*n* = 15 images per condition, at least 200 cells per sample were analyzed). Significance was tested by using the Mann–Whitney test with GraphPad software and refers to the control (^∗^*p* < 0.1, ^∗∗^*p* < 0.01, ^∗∗∗^*p* < 0.001, ^∗∗∗∗^*p* < 0.0001).

**FIGURE 2:**
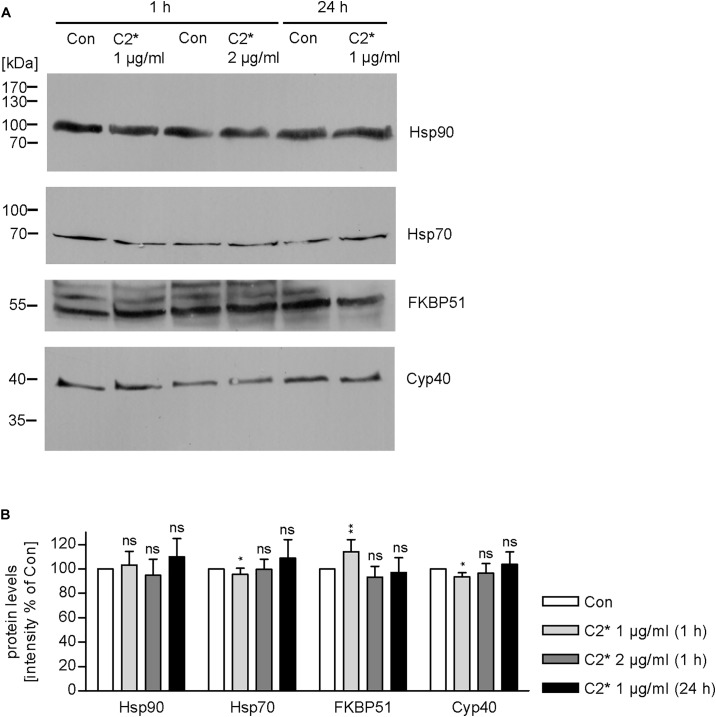
Expression levels of host cell factors in cells after C2^∗^ treatment. Vero cells were incubated with C2^∗^ toxin (C2I E387/389Q plus C2IIa, 1 or 2 μg/ml, each) for indicated time points or left untreated for control. After washing, cells were lysed in Laemmli sample buffer and subjected to SDS–PAGE and Western blotting. Ponceau S staining confirmed successful transfer to nitrocellulose membrane (not shown). After blocking, the membrane was cut and probed with specific antibodies against Hsp90, Hsp70, FKBP51, and Cyp40, and signals were detected by horseradish peroxidase coupled secondary antibodies using the ECL system **(A)**. **(B)** Intensity of Western blot signals was quantified by densitometry. Values are normalized to untreated control samples and are given as mean ± SD (*n* = 3 independent experiments). Significance was tested by using the student’s *t*-test with GrapPad software and refers to control samples (^∗^*p* < 0.1, ^∗∗^*p* < 0.01; ns, not significant).

Interaction between C2I and Hsp90, Cyp40, and FKBP51, respectively, has been analyzed and characterized *in vitro* in detail before (Kaiser et al., 2011, 2012; Ernst et al., 2015, 2017a). However, investigating single molecule interactions directly in cells has been a challenge due to detection limits of fluorescence microscopy. Here, by using PLA technology, which overcomes the detection limit in fluorescence microscopy for single molecule analysis by employing an internal amplification reaction, we demonstrate that C2I is in close proximity to Hsp90, Cyp40, and FKBP51 in living cells, implicating an interaction between C2I and these host cell factors (Figures 1A–D). Every gray dot signal shown in Figure 1 represents an interaction event between C2I and Hsp90, Cyp40, or FKBP51, respectively. As control, cells were not treated with C2 toxin and although a background level of interaction signals was observed, the number of PLA signals significantly increased in the presence of C2 toxin, which was most obvious in the quantitative analysis. Moreover, the interaction between C2I and Hsp90, Cyp40, or FKBP51 was concentration-dependent (Figure 1F) and not only demonstrated in Vero cells (Figures 1A–C) but in a second and human cell line, i.e., HeLa (Figure 1D). Moreover, Hsp90, Cyp40, and FKBP51 were visualized in the same experiment by using fluorescence-labeled secondary antibodies. Figure 1E shows that a comparable signal was obtained in samples treated with C2^∗^ toxin and control samples for Hsp90, Cyp40, and FKBP51 indicating no effect of C2^∗^ toxin treatment on protein levels of these host cell factors. We further analyzed protein levels of Hsp90, Hsp70, FKBP51, and Cyp40 by Western blotting confirming that C2^∗^ toxin treatment showed no relevant effects on the protein levels after 1 and 24 h (Figure 2). Previously, we demonstrated that Hsp70 is also required for the membrane translocation of C2I into the host cell cytosol and moreover employed PLA to demonstrate the interaction of Hsp70 with C2I. Therefore, we also assessed the protein levels of Hsp70 after C2^∗^ toxin treatment in this experiment.

**FIGURE 3:**
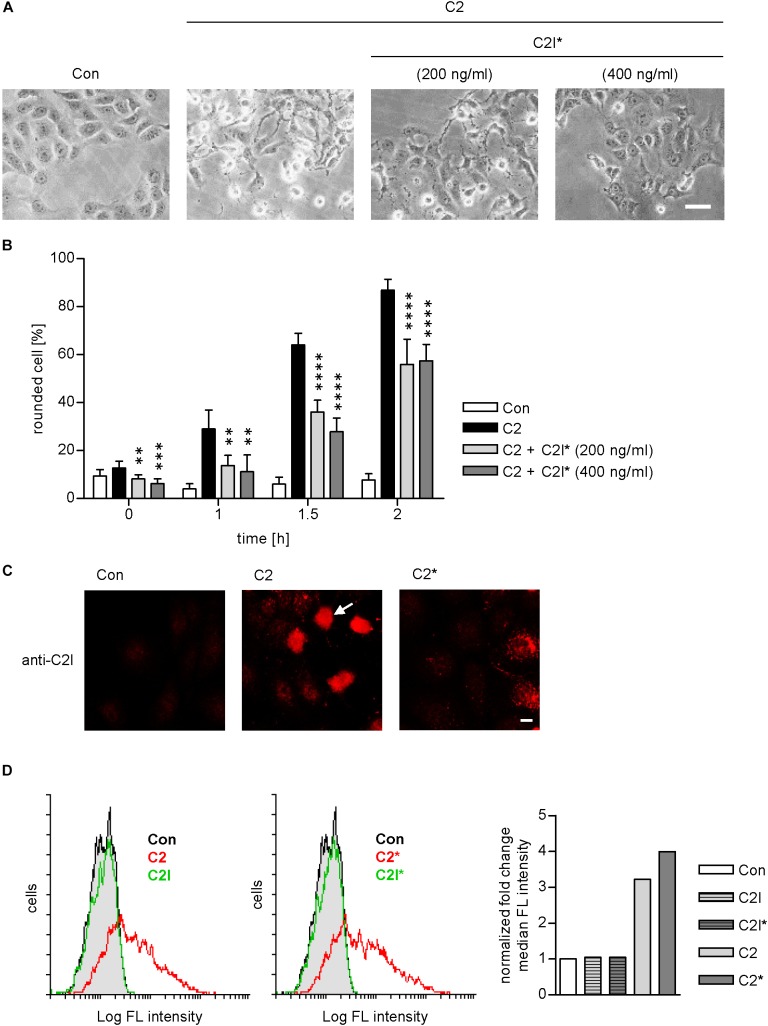
C2I E387/389Q competes with wild type C2I for uptake via C2IIa into cells. Vero cells were pre-incubated with C2 toxin (200 ng/ml C2I plus 400 ng/ml C2IIa) and with or without C2I E387/389Q (C2I^∗^) for 20 min on ice to allow binding to the membrane. Afterwards, cells were washed and incubated in toxin-free medium at 37°C to allow the toxin to enter the cell. **(A)** Representative pictures of cells 1.5 h after incubation at 37°C. Bar = 25 μm. **(B)** Percentage of rounded cells was determined from pictures taken at the indicated time points. Values are given as mean ± SD (*n* = 6 images from duplicates). The significance was tested with student’s *t*-test by GraphPad software and values refer to samples treated with C2 alone (^∗∗^*p* < 0.01, ^∗∗∗^*p* < 0.001, ^∗∗∗∗^*p* < 0.0001). **(C)** Vero cells were incubated with wildtype C2 or mutant C2^∗^ (0.5 μg/ml C2I or C2I E387/389Q, respectively, plus 1 μg/ml C2IIa) for 1 h. Cells were washed with PBS, fixed, permeabilized, and blocked with FCS. C2I or C2I E387/389Q were detected by a specific antiserum and fluorescence labeled secondary antibody. Bar = 10 μm. White arrow indicates rounded cell due to C2I enzyme activity. **(D)** C2 and C2^∗^ toxin bind to Vero cells. Vero cell suspensions (200,000 cells in 0.2 ml MEM containing serum) were incubated on ice for 10 min with DyLight488-labeled 0.6 μg/ml C2I or C2I E387/389Q (indicated as C2I^∗^) with or without 1.5 μg/ml C2IIa. After washing with PBS, cells were resuspended in medium and subjected to flow cytometry analysis. Results are presented as overlay histograms, where single cell events (cells) are plotted against the intensity of cell surface-bound fluorescence (Log FL intensity). Gray peak (identical in both overlays) represents the background fluorescence of mock-treated cells. The bar graph shows the fold change of the median fluorescence (FL) intensity of histogram peaks. Values are normalized to control. Results from one representative experiment are shown.

As indicated in Figure 1, the enzymatically inactive mutant C2IE387/389Q (Barth et al., 1998b) was used to perform the PLA assay. This mutant comprises two point mutations in the catalytic center of C2I preventing its ADP-RT activity and therefore cells treated with C2IIa plus C2IE387/389Q (referred to as C2^∗^) do not round up. This constitutes a clear advantage for the analysis of fluorescence microscopic images because signals, in particular PLA signals, are distributed more evenly in a flatter cell body and therefore can be detected more easily. Moreover, cells treated with C2^∗^ show stronger adherence to culture dishes than cells treated with the wild type C2 toxin and therefore are more resistant against the multiple washing steps required for PLA analysis. Earlier, we described that enzymatic inactivation of C2I does not alter its uptake properties via C2IIa, which is a prerequisite to use C2IE387/389Q for PLA experiments (Hilger et al., 2009). Here, we confirmed this biological activity of C2IE387/389Q by performing a competition assay. Cells were treated with C2IIa plus C2I in the presence of two different concentrations of C2IE387/389Q. Results in Figures 3A,B show that the intoxication of cells with wild-type C2 is significantly delayed if C2IE387/389Q is present demonstrating that C2IE387/389Q competes with wild type C2I for binding to C2IIa and therefore confirms biological activity of C2IE387/389Q. Since cell rounding due to the C2I-catalyzed ADP-ribosylation of G-actin in cytosol of cultured cells represents a highly sensitive endpoint of toxin uptake, a lower concentration of C2 toxin compared to fluorescence microscopy experiments can be used in this experimental setup. Flow cytometry analysis shows that wild type C2I and C2IE387/389Q bind to cells via C2IIa, and no binding was detected of enzyme components alone (Figure 3D). Fluorescence microscopy demonstrates that wild type C2I and C2IE387/389Q were taken up into cells (Figure 3C), which was most obvious in cells that were still flat and did not round up yet. In rounded cells, indicated by the white arrow, the signal accumulates on a smaller area and is therefore difficult to analyze. Hence, C2^∗^ was used for PLA experiments. Moreover, we earlier demonstrated exemplarily that binding of wild-type C2I and C2IE387/389Q to Cyp40 is comparable confirming suitability of C2IE387/389Q for PLA analysis (Ernst et al., 2015).

### Combined Pharmacological Inhibition of Host Cell Factors Has Stronger Effect in Delaying Toxin-Induced Cell Morphology Than Inhibition of Each Factor Alone

**FIGURE 4:**
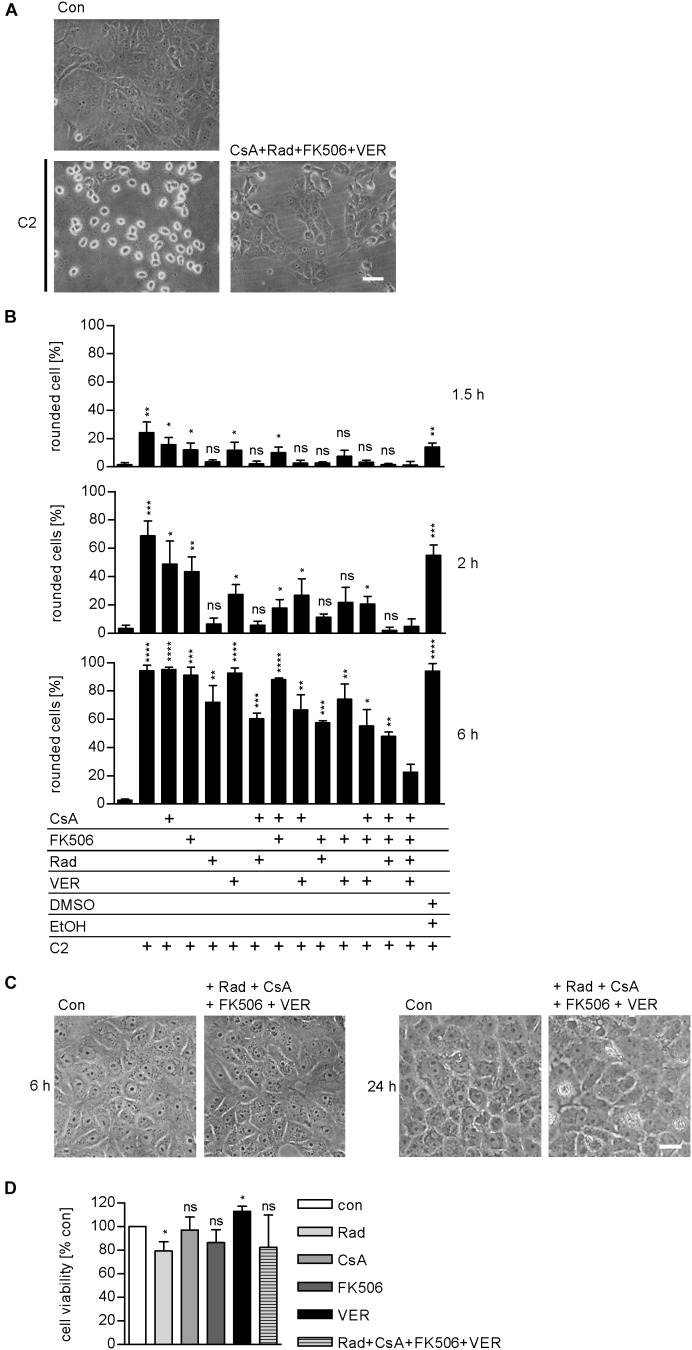
Combined inhibition of host cell factors delays toxin-induced cell morphology. Vero cells were pre-incubated with CsA (20 μM), FK506 (30 μM), Rad (20 μM), and VER (30 μM) alone or in combinations for 30 min at 37°C and then intoxicated with C2 toxin (25 ng/ml C2I plus 50 ng/ml C2IIa) and incubated at 37°C. For control, cells were left untreated, treated with C2 toxin only or treated with solvents of inhibitors (DMSO + EtOH) and C2 toxin. **(A)** Pictures show the morphological changes of cells 6 h after toxin treatment. Con, untreated cells. Bar = 50 μm. **(B)** Percentage of rounded cells was determined from images at indicated time points. Values are given as mean ± SD (*n* = 3). Significance was tested with Student’s *t*-test by GraphPad software and values refer to samples treated with C2 toxin plus combination of Rad, CsA, FK506, and VER for each time point (ns, not significant; ^∗^*p* < 0.1, ^∗∗^*p* < 0.01, ^∗∗∗^*p* < 0.001, ^∗∗∗∗^*p* < 0.0001). **(C)** Vero cells were incubated with a combination of Rad, CsA, FK506, and VER or left untreated for control. Pictures were taken after 6 and 24 h. Bar = 50 μm. **(D)** Vero cells were incubated with a combination of Rad, CsA, FK506, and VER or left untreated for control. After 24 h, cell viability was measured by adding MTS reagent for 1 h at 37°C and resulting formazan was measured at 490 nm. Values are given as mean ± SD and are normalized to untreated control (*n* = 3 independent experiments). Significance was tested by using the student’s *t*-test with GraphPad software and refers to control samples (^∗^*p* < 0.1; ns, not significant).

We demonstrated and characterized the protective effects of the single application of 1–50 μM radicicol [Rad, Hsp90 inhibitor (Pratt and Toft, 2003)], 10–20 μM cyclosporine A [CsA, Cyp inhibitor (Borel et al., 1976)], 20 μM FK506 [FKBP inhibitor (Harding et al., 1989)], and 30 μM VER [Hsp70 inhibitor (Williamson et al., 2009)] on cells in detail during the last years (Haug et al., 2003a, 2004; Kaiser et al., 2009, 2011, 2012; Ernst et al., 2015, 2016, 2017a). Combinations of two inhibitors, for example, the combination of 1 μM Rad with 20 μM CsA or 10 μM CsA with 20 μM FK506, have been tested and proven to result in a greater inhibition of intoxication with C2 toxin (Kaiser et al., 2009, 2011, 2012). However, a combination of all four inhibitors has not been tested so far. Here, we show that pharmacological inhibition of Hsp90, Hsp70, Cyps, and FKBPs results in a significantly stronger inhibition of intoxication of cells with C2 toxin compared to inhibition of single, two, or three host cell factors (Figure 4). This effect became most obvious after longer incubation periods, i.e., 6 h. Notably, application of Rad, CsA, FK506, and VER in combination had no relevant effects on cell morphology after 6 and 24 h or cell viability after 24 h (Figures 4C,D).

### Combined Pharmacological Inhibition of Host Cell Chaperones/PPIases Prevents Membrane Translocation of C2I Into the Cytosol

**FIGURE 5:**
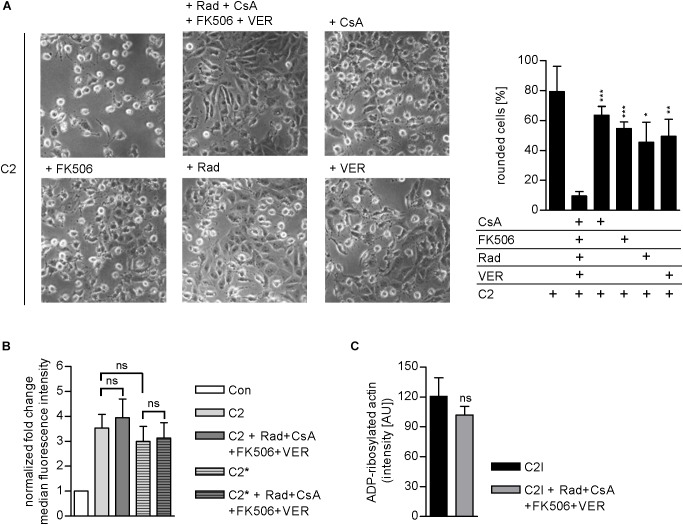
**(A)** Combined inhibition of host cell chaperones/PPIases prevents membrane translocation of C2I into cytosol. Vero cells were pre-incubated with CsA (20 μM), FK506 (30 μM), Rad (20 μM), and VER (30 μM) alone or in combination for 30 min at 37°C in BafA1-containing medium. As control, cells were treated with BafA1-containing medium only. Cells were cooled to 4°C, and C2 toxin was added on ice for 20 min. Then, cells were challenged with warm acidic medium for 5 min at 37°C. For control, cells were treated with neutral medium (not shown). Medium was exchanged to fresh pH neutral medium containing BafA1 and respective inhibitors and cells were further incubated for 2.5 h. Toxin-induced morphological changes were monitored. Bar = 50 μm. Percentage of rounded cells was determined from pictures. Values are given as mean ± SD (*n* = 3 pictures per sample). Results from one representative experiment are shown. Significance was tested with Student’s *t*-test by GraphPad software and values refer to samples treated with C2 toxin plus combination of Rad, CsA, FK506, and VER (ns, not significant; ^∗^*p* < 0.1, ^∗∗^*p* < 0.01, ^∗∗∗^*p* < 0.001). **(B)** Effect of the combination of Rad, CsA, FK506, and VER on the binding of C2 or C2^∗^ toxin to Vero cells. Vero cell suspensions (200,000 cells in 0.2 ml MEM containing serum) were incubated with CsA (20 μM), FK506 (30 μM), Rad (20 μM), and VER (30 μM) or left untreated for 30 min at 37°C. Subsequently, cells were incubated on ice for 5 min and then further incubated with DyLight488-labeled 0.6 μg/ml C2I or C2I E387/389Q (C2I^∗^) plus 1.5 μg/ml C2IIa for 10 min on ice. After washing with PBS, cells were resuspended in medium and subjected to flow cytometry analysis. The median FL intensity was calculated from histograms. Shown is the fold change of the median FL intensity normalized to control (*n* = 6 values from two independent experiments). Values are given as mean ± SD. Significance was tested with Student’s *t*-test by GraphPad software (ns, not significant). **(C)** Effect of the combination of Rad, CsA, FK506, and VER on the enzyme activity of C2I *in vitro*. Vero cell lysate (20 μg) was pre-incubated with CsA (20 μM), FK506 (30 μM), Rad (20 μM), and VER (30 μM) or left untreated for 30 min at 37°C. C2I (200 ng) and biotin-NAD^+^ were added for 20 min at 37°C. Biotin-labeled i.e., ADP-ribosylated actin was detected via streptavidin-coupled horseradish peroxidase using the ECL system. Intensity of Western blot signals was quantified by densitometry. Values are given as mean ± SD (*n* = 4). Results from one representative experiment are shown. Significance was tested by using the student’s *t*-test with GraphPad (ns, not significant).

Results from previous studies concerning the effect of single application of chaperone/PPIase inhibitors suggest that the combination of Rad, CsA, FK506, and VER interferes with the membrane translocation of C2I from early endosomes into the cytosol of target cells. To elucidate which step of toxin uptake/mode of action is affected by the combined pharmacological inhibition of Hsp90, Hsp70, Cyps, and FKBPs, we analyzed the membrane translocation of C2I in an isolated manner. Therefore, the membrane translocation step was mimicked directly at the cytoplasmic membrane to enable analysis of this step of toxin uptake in an isolated manner. To this end, cells were incubated on ice to minimize membrane fluidity and thus endocytosis. BafA1 is an inhibitor of the v-ATPase and was added to inhibit the “normal” uptake route of C2I over acidified endosomes (Sandvig and Olsnes, 1980; Bowman et al., 1988). After binding of C2 toxin to the cooled cells, warm acidic medium was added to mimic acidic conditions in endosomes and thus enable pore formation by C2IIa and translocation of C2I directly through the cytoplasmic membrane into the cytosol of cells. Rounding of adherent cells was employed as a specific endpoint of toxin uptake in this assay. In this assay too, the combination of Rad, CsA, FK506, and VER revealed a stronger inhibitory effect than application of the single substances and efficiently protected cells from intoxication with C2 toxin (Figure 5). Importantly, the combination of Rad, CsA, FK506, and VER did not significantly inhibit other steps of toxin uptake or C2I enzyme activity (Figures 5B,C). This result confirms that combined inhibition of Hsp90, Hsp70, Cyps, and FKBPs interferes with the membrane translocation of C2I from early endosomes into the cytosol of target cells.

Taken all together, we showed that Hsp90, Cyp40, and FKBP51 are located in close proximity with C2I in Vero and HeLa cells and therefore most likely interact in the cytosol of target cells. This interaction was concentration-dependent. Protein levels of Hsp90, Hsp70, FKBP51, and Cyp40 were not affected by C2^∗^ toxin treatment. Moreover, we demonstrated that the combination of specific pharmacological inhibitors of chaperone/PPIase activities protected target cells from intoxication with C2 toxin and interfered with membrane translocation of C2I from endosomes into the cytosol but not with cell binding or enzyme activity. Interestingly, the effects of the combination of Hsp90, Hsp70, Cyp, and FKBP activity inhibition were significantly stronger than the effects of a single inhibitor treatment and combining Rad, CsA, FK506, and VER had no significant effects on cell viability after 24 h. This finding might be of interest regarding the development of novel pharmacological strategies against diseases caused by toxin-producing bacteria.

## Discussion

Bacterial AB-type protein toxins are the causative agents for various severe diseases, such as diphtheria, whooping cough, cholera, and further enteric diseases. Although several of the toxin-induced diseases have been known for a long time and therapeutic or vaccination strategies are available, these diseases pose a severe threat to human and also livestock health and survival. The enterotoxin *C. botulinum* C2 toxin consists of two components: C2I harboring ADP-ribosyltransferase activity and C2IIa facilitating receptor-binding, uptake, and translocation of C2I into the cytosol of target cells. Here, C2I covalently transfers an ADP-ribose moiety onto G-actin leading to depolymerization of F-actin and therefore to rounding up of adherent cells. C2 has been shown to display severe enterotoxicity in various animal models (Barth and Ernst, 2016; Stiles, 2017). A necessity for C2 toxin to exert this enterotoxicity is to reach the cytosol of target cells since there its substrate G-actin is localized. C2 toxin employs a very elaborate uptake mechanism during, which cellular protein trafficking mechanisms are exploited, i.e., receptor-binding or receptor-mediated endocytosis. A particularly crucial step of toxin uptake is the membrane translocation from endosomal compartments to the cytosol. The physiologically acidification of the endosomal lumen leads to conformational changes in both components of C2 toxin (Barth et al., 2000; Haug et al., 2003b). The C2IIa heptamer forms a pore into the endosomal membrane and C2I is at least partially unfolded to translocate through the narrow C2IIa pores into the cytosol. During the last years, we discovered that the C2 toxin exploits further cellular mechanisms for successfully reaching the cytosol: C2I interacts with host cell chaperones and PPIases, which facilitate its translocation and most likely its refolding into a native and therefore enzymatically active conformation (Barth and Ernst, 2016; Ernst et al., 2017b). This suggests that the chaperones and PPIases should represent attractive novel drug targets in the context of diseases that are caused by such bacterial protein toxins. Indeed, inhibition of the activity of Hsp90, Hsp70, Cyps, and FKBPs by specific pharmacological inhibitors protected cells from intoxication with C2 toxin and specifically inhibited the membrane translocation of C2I into the cytosol. These findings harbor medical implications because if C2I cannot reach the cytosol G-actin cannot be modified and clinical symptoms can be prevented.

Here, we show that C2I is in close proximity and therefore most likely interacting with Hsp90, Cyp40, and FKBP51 in cells indicating their importance for successful toxin uptake into the cell. *In vitro*, this interaction has been demonstrated and characterized in detail before. Hsp90, Cyp40, and FKBP51 co-precipitated with C2I from toxin-treated cells (Kaiser et al., 2012; Ernst et al., 2015). A direct interaction between Hsp90, Cyp40, FKBP51, and C2I was demonstrated using dot blot analysis (Kaiser et al., 2011, 2012; Ernst et al., 2015, 2016, 2017a). Moreover, we exemplarily determined the dissociation constant of the C2I–Cyp40 interaction using isothermal titration calorimetry to be in the nanomolar range (*K*_D_ = 101 nM) (Ernst et al., 2015). Analysis of an interaction in cells harbors difficulties since the detection of single molecule interaction by fluorescence microscopy challenges its detection limit. In our previous work, we established a novel assay, the PLA, and thereby demonstrated the interaction between Hsp70 and C2I in cells (Ernst et al., 2017a). This assay allows the detection of two molecules in close proximity via fluorescence microscopy by employing an internal signal amplification reaction that is polymerase-based (Söderberg et al., 2008). Here, this PLA method was used to show that also Hsp90, Cyp40, and FKBP51 were in close proximity i.e., interacted with C2I in C2-treated cells. This further confirms the important role these host cell factors play during toxin uptake.

The activity of Hsp90, Hsp70, Cyps, and FKBPs can be prevented by specific pharmacological inhibitors. Rad binds to the ATP-binding pocket of Hsp90 thereby inhibiting its activity in cells (Pratt and Toft, 2003; Li and Buchner, 2013). The same mechanism holds true for inhibition of Hsp70 by VER (Williamson et al., 2009). CsA and FK506 interfere with PPIase activity of Cyps and FKBPs, respectively (Handschumacher et al., 1984; Fischer et al., 1989; Harding et al., 1989; Galat, 2003). Previously, we demonstrated that Rad, VER, CsA, and FK506 protect cells from intoxication of C2 toxin and combinations of two inhibitors resulted in stronger protection of cells compared to application of single substances (Haug et al., 2003a; Kaiser et al., 2009, 2012; Ernst et al., 2015, 2017a). The present study reveals that a combination of Rad, VER, CsA, and FK506 has an even stronger inhibitory effect than the single inhibitors or combinations of two on intoxication and in particular membrane translocation of C2I to the cytosol. One could speculate that the molecular mechanisms underlying the observed stronger protection of cells by a combination of Hsp90, Hsp70, Cyp, and FKBP inhibition compared to an application of the single substances may arise from the fact that targeted folding helper proteins with similar functions in supporting/promoting protein folding are able to functionally replace each other in cells. Thus combination of the different drugs could circumvent partial or even nearly complete rescue by the folding helper proteins still in function. It might be that the pharmacological inhibition of one folding helper enzyme generates conditions in the cells, under which the presence of one or more of the other folding helpers are required to maintain cellular life. In case of a steroid receptor complex like scenario, the stronger effects of combined inhibition may also result from functional replacement. There, it could also be that only the complete inhibition of such a complex occurs, if all the components of the complex are inhibited and that incomplete complexes are functional, but in a diminished manner or not with their full efficiency.

At the same time, the combination of inhibitors alone had no adverse effects on the morphology of cells. Rad and derivatives have been tested in anti-tumor treatment, revealing, however, some adverse effects that require the search and development for novel well-tolerated derivative (Li and Buchner, 2013). CsA and FK506 are approved immunosuppressive drugs applied mostly after organ transplantation to prevent organ rejection (Borel et al., 1976; Liu et al., 1991; Schreiber et al., 1991). Interaction of CsA or FK506 with Cyps or FKBPs, respectively, allows the inhibitor-protein complex to interact with a protein phosphatase in cells, calcineurin. This leads to inhibition of calcineurin and therefore the transcription factor nuclear factor of activated T-cells (NF-AT) cannot be dephosphorylated by calcineurin resulting in decreased activation of T-lymphocytes (Liu et al., 1991; Schreiber et al., 1991; Clipstone and Crabtree, 1992). Although this mode of action is necessary to use CsA/FK506 as immunosuppressant, in the context of a bacterial infection administration of an immunosuppressive drug is highly counterproductive. In 2015, we demonstrated that a CsA derivative, VK112, which lacks the immunosuppressive effect but still inhibits PPIase activity of Cyps, protects cells from intoxication with the clostridial C2, iota, and CDT toxin (Prell et al., 2013; Ernst et al., 2015), and we further extended these findings for the diphtheria toxin (Schuster et al., 2017) and pertussis toxin (Ernst et al., 2018). Therefore, the development and engineering of pharmacological inhibitors are highly demanded and represent a promising opportunity to discover novel therapeutic strategies against diseases caused by bacterial protein toxins.

This is of special importance since the current treatment options for several toxin-caused diseases are limited. For example, there is no causative treatment available against the severe childhood disease whooping cough, caused by pertussis toxin produced by *Bordetella pertussis*, which can be life-threatening especially to newborns that cannot be vaccinated yet (Mattoo and Cherry, 2005; Carbonetti, 2015; World Health Organization [WHO], 2016). Moreover, *C. difficile* infections pose a severe threat especially to hospitalized patient undergoing antibiotic therapy (Aktories et al., 2018; Papatheodorou et al., 2018). Case numbers are still increasing every year in Western countries and hypervirulent strains are emerging. The two AB-type toxins A and B of *C. difficile* cause severe symptoms such as antibiotic-associated diarrhea and pseudomembranous colitis (Aktories et al., 2017a). Hypervirulent *C. difficile* strains produce a third toxin, CDT, additionally to toxins A and B, which belongs to the ADP-ribosylating toxins. CDT increases the adherence of *C. difficile* in the gut by forming long protrusions in which the bacteria get caught. For these strains, an increased reoccurrence of the infection has been reported, increasing the risk for developing life-threatening complications, and a prolonged suffering for the patient (Aktories et al., 2018). Up to now, CDI is treated with specific antibiotics and an antibody against toxin B is available. However, antibodies or antitoxins can only eliminate toxin molecules that did not bind to cells or have been internalized yet. Therefore, novel therapeutic strategies i.e., targeting chaperones/PPIases that act intracellular might be a valuable asset to improve and support current treatment strategies to prevent severe courses of disease and reoccurrence of CDI.

During our investigations over the last years, we observed that all toxins we investigated so far which harbor an ADP-ribosyltransferase activity require chaperones and PPIases for their membrane translocation regardless of their structure, composition, or uptake route into the cell (Ernst et al., 2017b). This group of toxins comprises the clostridial binary toxins, diphtheria toxin, pertussis toxin, cholera toxin, and the large insecticidal toxins PTC3 and PTC5 (Haug et al., 2003a, 2004; Kaiser et al., 2009, 2011, 2012; Burress et al., 2014; Lang et al., 2014; Ernst et al., 2015, 2016, 2017a, 2018; Schuster et al., 2017). Toxins displaying a different enzyme activity such as the *C. difficile* glucosylating toxins or *Bacillus anthracis* lethal toxin are independent of these host cell factors (Haug et al., 2003a; Kaiser et al., 2009, 2011; Zornetta et al., 2010; Dmochewitz et al., 2011). The uptake of toxin constructs artificially containing an ADP-ribosyltransferase activity or isolated ADP-ribosyltransferase domains relies on the activity of Hsp90, Hsp70, Cyps, and FKBPs (Haug et al., 2003a; Dmochewitz et al., 2011; Lang et al., 2014; Ernst et al., 2017a). This implicates that novel therapeutic strategies developed on the basis of these chaperones/PPIases might not only be used to treat one disease, but all diseases caused by the same group of bacterial toxins, namely the ADP-ribosylating toxins.

## Author Contributions

KE designed, supervised and conducted the experiments and wrote the manuscript. CK and ML conducted the experiments. HB designed and supervised the study and wrote the manuscript.

## Conflict of Interest Statement

The authors declare that the research was conducted in the absence of any commercial or financial relationships that could be construed as a potential conflict of interest.
